# Anecdote Regarding Lord Lister

**Published:** 1897-06

**Authors:** 


### Anecdote Regarding Lord Lister.

While going round his wards in the Glasgow Royal Infirmary one day, Sir Joseph, then plain Mr. Lister, came to the bedside of a patient whose arm had been severely crushed without the skin having received any injury. Turning to the assembled students, he said: "Gentlemen, I have frequently noticed that when severe injuries are received without the skin being broken, the cases nearly always recover. On the other hand, trouble is always apt to follow, even in trivial injuries, when a wound in the skin is present. How is this? I can not help thinking that the man who is able to explain this problem will be one who will gain for himself undying fame." Lord Lister himself has proved the truth of his prophecy.—*London Daily News.*

